# Insights into Natural History, Phenotypic, and Molecular Spectrum in a Large Cohort of Osteosclerotic Disorders

**DOI:** 10.1007/s00223-025-01366-w

**Published:** 2025-04-08

**Authors:** Dilek Uludağ Alkaya, Esra Usluer, Zeynep Alp Ünkar, Ali Şeker, İbrahim Adaletli, Nilay Güneş, Rıza Madazlı, Pınar Kadıoğlu, Murat Derbent, Beyhan Tüysüz

**Affiliations:** 1 Department of Pediatric Genetics, Cerrahpaşa Medical Faculty, Istanbul University-Cerrahpaşa, Istanbul, Turkey; 2 Department of Neonatology, Cerrahpaşa Medical Faculty, Istanbul University-Cerrahpaşa, Istanbul, Turkey; 3 Department of Orthopedics and Traumatology, Cerrahpaşa Medical Faculty, Istanbul University-Cerrahpaşa, Istanbul, Turkey; 4 Department of Pediatric Radiology, Cerrahpaşa Medical Faculty, Istanbul University-Cerrahpaşa, Istanbul, Turkey; 5 Department of Perinatology, Cerrahpaşa Medical Faculty, Istanbul University-Cerrahpaşa, Istanbul, Turkey; 6 Department of Internal Medicine, Endocrinology, Cerrahpaşa Medical Faculty, Istanbul University-Cerrahpaşa, Istanbul, Turkey; 7 Department of Pediatric Genetics, Medical Faculty, Başkent University, Ankara, Turkey

**Keywords:** Osteosclerotic disorders, *ANKH*, *SOST*, *TNFRSF11B*, *TBXAS1*, *HPGD*

## Abstract

Osteosclerotic bone diseases include more than 30 rare diseases characterized by excessive bone formation. The aim of this study is to compare the molecular pathogenesis and prognostic features of 12 different osteosclerotic diseases. Thirty-four patients from 23 families were included, 25 of whom were followed for a period of one to 22 years. Exome sequencing was performed in 20 families. Primary hypertrophic osteoarthropathy (PHOAR1/2) was found in 12 patients, followed by juvenile Paget’s disease (JPD)-5 in five, craniometaphyseal dysplasia (CMD) and Camurati-Engelmann disease (CED) in four, Ghosal hematodiaphyseal dysplasia (GHDD) in three patients, sclerosteosis-1 in two patients, and ultra-rare diseases including trichothiodystrophy-1, prenatal Caffey disease, melorheosteosis, and Lenz-Majewski hyperostotic dwarfism in one patient each. Patients with CMD and sclerosteosis-1 had severe cranial sclerosis leading to facial dysmorphism. CMD was characterized by metaphyseal widening, radiolucency, and diaphyseal sclerosis of the long bones in early childhood and later developed Erlenmeyer flask deformity sparing the vertebrae and pelvis, whereas sclerosteosis-1 manifested as generalized sclerosis. CED and GHDD share bone pain, difficulty in walking, and diaphyseal sclerosis, with some patients also having bone marrow involvement. Interestingly, patients with CED and JPD-5 showed osteopenia in early childhood, followed by the development of osteosclerosis in late childhood. Clinical and radiologic findings improved over time in PHOAR1 patients, whereas they progressed in JPD-5 and trichothiodystrophy-1 patients. Intra- and interfamilial clinical differences were observed in CMD, CED, JPD-5, and GHDD. The knowledge gained about the natural history of osteosclerotic diseases will make an important contribution to their diagnosis and management.

**Supplementary Information:**

The online version contains supplementary material available at 10.1007/s00223-025-01366-w.

## Introduction

Sclerosing bone dysplasias are a heterogeneous group of disorders characterized by increased bone density [1, 2]. In the 2023 revised nosology of genetic skeletal disorders, these conditions are categorized into two main groups based on the underlying pathogenic mechanism: Osteopetrosis and related osteoclast disorders, and osteosclerotic disorders [3]. Osteopetrosis and related osteoclastic disorders (group 24) result from impaired bone resorption due to defects in the number or function of osteoclasts while osteosclerotic disorders are mainly caused by excessive bone formation or disruptions in bone remodeling [1]. Osteosclerotic disorders, classified as group 25, combine the previously distinct subcategories of neonatal osteosclerotic dysplasias and other sclerosing bone disorders [3]. This group encompasses various diseases, including sclerosteosis-1, Juvenile Paget’s disease (JPD)-5, Ghosal hematodiaphyseal dysplasia (GHDD), primary hypertrophic osteoarthropathy (PHOAR) types 1 and 2, caused by biallelic mutations in *SOST/LRP4, TNFRSF11B, TBXAS1*, and *HPGD/SLCO2A1,* as well as craniometaphyseal dysplasia (CMD) and Camurati-Engelmann disease (CED), caused by monoallelic mutations of *ANKH* and *TGFB1,* respectively [1–3]. Extremely rare osteosclerotic disorders also include Caffey disease, Lenz-Majewski hyperostotic dwarfism, trichothiodystrophy-1 with axial osteosclerosis, and melorheostosis which are caused by mutations in *COL1A1, PTDSS1*, *ERCC2*, and *MAP2K1,* respectively [4–6].

Several signaling pathways such as WNT, TGF-β, RANK/nuclear factor-kappa B (NFκB), and impaired prostaglandin metabolism and pyrophosphate homeostasis play a pivotal role in the pathogenesis of osteosclerotic disorders [7–14]. The clinical spectrum of osteosclerotic disorders is very heterogeneous and ranges from severe conditions that reduce life expectancy to mild skeletal abnormalities. There are few studies investigating the detailed clinical findings and prognostic features of the different types. The aim of this study is to investigate the underlying genetic etiology of osteosclerotic disorders and to compare the clinical features and prognostic outcomes in a large Turkish cohort.

## Methods

### Subjects

The study group consisted of 34 cases from 23 unrelated families with osteosclerotic disorders. This group was selected from the main group of 67 sclerotic bone disease patients, referred over the past 25 years to a center with experience in skeletal dysplasia. Those with osteopetrosis and related osteoclastic disorders and with secondary sclerotic bone disease were excluded. Three families with previously published molecular and clinical findings were included in this study in order to evaluate follow-up findings [15–17]. Physical examinations were performed at the time of diagnosis and at annual follow-up visits, including assessment of growth parameters, joint mobility, neurological examination, and ophthalmologic, hearing and dental examinations. In addition, skeletal radiographs, bone mineral density (BMD) and biochemical parameters were assessed. The annual incidence of fractures was documented. BMD measurements of the lumbar vertebrae L1–L4 were performed using dual-energy X-ray absorptiometry (DEXA) with results expressed as Z-scores. Developmental delay and intellectual disability were assessed using the Denver Developmental Screening Test II or the WISC-R.

### Genetic Studies

Exome sequencing was performed on one patient from each of the 20 unrelated families using the QIAseq Human Exome Kit followed by 101 base paired-end sequencing on the Illumina NovaSeq instrument. Sequences were aligned to the human genome GRCh38 with BWA-MEM. Variants were identified using GATK Haplotype Caller and annotated with ANNOVAR. Variants with high-quality sequence reads, a genotype quality ≥ 20, and minor allele frequency ≤ 10^–2^ across all samples in gnomAD were included. These variants were classified according to the American College of Medical Genetics and Genomics guidelines. Segregation analysis was performed on each family using Sanger sequencing. The identified mutation was confirmed in the proband and other affected family members, and carrier status was assessed in the parents for biallelic variants. Sanger sequencing was performed using an ABI PRISM 3500 genetic analyzer (Applied Biosystems, Foster City, CA, USA). The data were analyzed using the Cutepeaks software (https://github.com/labsquare/cutepeaks).

The genetic analysis could not be performed in three patients who were clinical diagnosed with Caffey, Lenz-Majewski hyperostotic dwarfism, and primary hypertrophic osteoarthropathy-1 diseases, due to lack of available genetic testing methods at the time of diagnosis.

## Results

A total of 34 patients (one mother, ten siblings, and one fetus) from 23 unrelated families were evaluated. Twenty five of these patients were followed for a duration ranging from one to 22 years. Consanguineous marriages were identified in nine of the families (39%).

### Molecular Features

Molecular etiology was identified in 19 of the 20 families. No pathogenic variant was detected in a patient with melorheostosis. A total of 15 disease-causing variants, including five novel variants, were discovered in eight genes: Monoallelic variants in *ANKH* and *TGFB1*, and biallelic variants in *SOST*, *TBXAS1*, *TNFRSF11B*, *HPGD*, *SLCO2A1*, and *ERCC2* (Table 1).

**Table 1:** Molecular findings of the families with osteosclerotic disorders

Family/Patient number	Clinical Diagnosis	Gene (Transcript)	Zygosity	Variant (ACMG classification)	Protein Change
F1/P1	CMD	*ANKH* (NM_054027)	Monoallelic	c.1124_1126del (LP)	p.Ser375del
F2/P2–P4	CMD	*ANKH* (NM_054027)	Monoallelic	c.1145C > G (VUS)	p.Thr382Arg (Novel)
F3/P5, P6	Sclerosteosis-1	*SOST* (NM_025237)	Biallelic	c.372G > A (P)	p.Trp124*
F4/P7	CED	*TGFB1* (NM_000660)	Monoallelic	c.652C > T (P)	p.Arg218Cys
F5/P8	CED	*TGFB1* (NM_000660)	Monoallelic	c.652C > T (P)	p.Arg218Cys
F6/P9	CED	*TGFB1* (NM_000660)	Monoallelic	c.652C > T (P)	p.Arg218Cys
F7/P10	CED	*TGFB1* (NM_000660)	Monoallelic	c.652C > T (P)	p.Arg218Cys
F8/P11	GHDD	*TBXAS1* (NM_030984)	Biallelic	c.1235G > A (LP)	p.Arg412Gln
F9/P12, P13	GHDD	*TBXAS1* (NM_030984)	Biallelic	c.748G > T (LP)	p.Glu250* (Novel)
F10/P14, P15	JPD-5	*TNFRSF11B* (NM_002546)	Biallelic	c.814C > T (LP)	p.Gln272* (Novel)
F11/P16, P17	JPD-5	*TNFRSF11B* (NM_002546)	Biallelic	c.2 T > C (LP)	p.Met1Thr (Novel)
F12/P18*	JPD-5	*TNFRSF11B* (NM_002546)	Biallelic	c.193C > T (LP)	p.Cys65Arg
F13/P19, P20*	PHOAR-1	*HPGD* (NM_000860)	Biallelic	c.310_311del (P)	p.Leu104Alafs*3
F14/P21	PHOAR-1	*HPGD* (NM_000860)	Biallelic	c.175_176del (P)	p.Leu59Valfs*8
F15/P22, P23	PHOAR-1	*HPGD* (NM_000860)	Biallelic	c.418G > C (P)	p.Ala140Pro
F16/P24, P25	PHOAR-1	*HPGD* (NM_000860)	Biallelic	c.310_311del (P)	p.Leu104Alafs*3
F17/P26	PHOAR-1	*HPGD* (NM_000860)	Biallelic	c.418G > C (P)	p.Ala140Pro
F19/P28, P29, P30*	PHOAR-2	*SLCO2A1* (NM_005630)	Biallelic	c.830dup (P)	p.Phe278Leufs*18
F20/P31	Trichothiodystrophy	*ERCC2* (NM_000400)	Biallelic	c.[2088G > T]; (VUS)/c.[423dup] (LP)	p.[Trp696Cys] (Novel); p.[Val142Cysfs*8]

^*^These patients were reported previously (Chong et al., [15]; Seifert et al., [16]; and Tüysüz et al., [17]). CED: Camurati-Engelmann disease,

CMD: Craniometaphyseal Dysplasia, GHDD: Ghosal Hematodiaphyseal Dysplasia, JPD: Juvenile Paget Disease, PHOAR: Primary Hypertrophic Osteoarthropathy, P: Pathogenic, LP: Likely Pathogenic, VUS:Variant of Uncertain Significance

### Clinical and Radiologic Features

Based on the clinical and radiologic evaluations, nine patients were diagnosed with primary hypertrophic osteoarthropathy type 1, three with primary hypertrophic osteoarthropathy type 2, five with Juvenile Paget’s disease-5, four with craniometaphyseal dysplasia, four with Camurati-Engelmann disease, three with Ghosal hematodiaphyseal dysplasia, two with sclerosteosis-1, one with trichothiodystrophy-1, one with Lenz-Majewski hyperostotic dwarfism, one with Caffey disease, and one with melorheostosis. The clinical features of the patients according to the different phenotypes are compared in Table S1. The detailed clinical and radiological features are summarized in Table S2.

### Craniometaphyseal Dysplasia

**Fig. 1:**
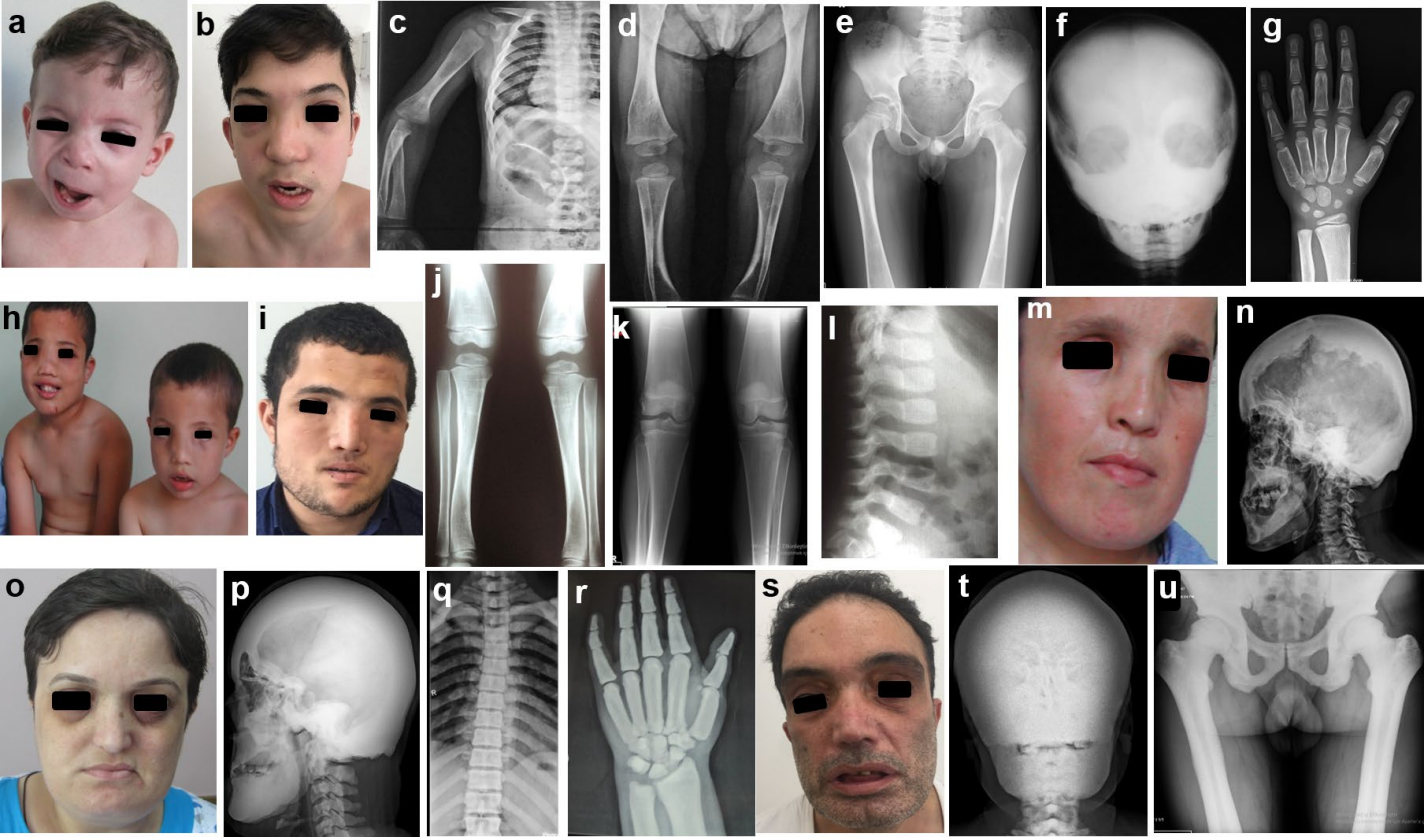
Four patients with craniometaphyseal dysplasia: P1 at the age of 18 months (**a**, **c**, **d**, **f**), 12 (**b**) and 8 years **(e, g)**. P2 at the age of 8.5 and 22 years (**h**, **i**). P3 at the age of 5.5 (**h**, **j**, **l**) and 16 years (**k**). Facial palsy and typical facial features including long face, broad nasal bridge, hypertelorism, midface hypoplasia, and prognathism were revealed on photographs (**a**, **b**, **h**, **i**). P4 had mild dysmorphic face at the age of 44 years (**m**). Cranial radiographs of P1 (**f**) and P4 (**n**) reveal diffusely sclerotic calvarium with obliteration of sinuses. Radiographs of long bones show widening and radiolucency of metaphyses and diaphyseal sclerosis of long bones during early childhood (**c**, **d**, **j**). Note diaphyseal sclerosis has resolved over time (**k**). No sclerosis on pelvis, hand, and vertebrae graphies (**e**, **g**, **l**). P5 and P6 with sclerosteosis-1, at the age of 37 (**o**–**r**) and 45 years (**s**–**u**), respectively. Long, flat face, broad forehead, prognathism, and macrocephaly (**o**, **s**), diffuse cranial sclerosis (**p**, **t**) and significant hyperostosis on the vertebrae, pelvis, and tubular bones (**q**, **r**, **u**)

A recurrent monoallelic variant c.1124_1126del (p.Ser375del) in *ANKH* was detected in the P1, and a novel c.1145C > G (p.Thr382Arg) variant in P2, P3 and their mother (P4). Three children, aged between 1.4 to 5.5 years, were admitted, all presenting with facial dysmorphism, and one also had facial palsy (Fig. 1a, h). During follow-up, until the age of 16–25 years (Fig. b, i), all developed hearing loss. Radiographs showed diffuse progressive hyperostosis of cranial bones, and paranasal sinuses (Fig. 1f), widening, and radiolucency of the metaphyses and diaphyseal sclerosis of the long bones in early childhood (Fig. 1c, d, j), while the vertebra, pelvis, and hand showed normal densities (Fig. 1e, g, l). An Erlenmeyer flask deformity developed in late childhood and radiolucency of the metaphyses of the long bones resolved with age (Fig. 1k). Although the 40-year-old mother had severe sclerosis of the cranial bones (Fig. 1n), she showed no symptoms other than mild facial dysmorphism and mild hearing loss (Fig. 1m).

### Sclerosteosis-1

Two adult siblings (P5 and P6) carrying a biallelic recurrent variant c.372G > A (p.Trp124*) in *SOST* presented with macrocephaly, mild intellectual disability, hearing loss, facial dysmorphism, and partial syndactyly between third and fourth fingers (Fig. 1o, s). P5 also had facial paralysis, optic atrophy, and vision loss. Radiographs in both patients showed severe sclerosis of the cranial bones, broad and sclerotic clavicles and ribs, sclerotic vertebrae, pelvis, hand and long bones, thickened cortices, and scoliosis (Fig. 1p–r, t, u).

### Camurati-Engelmann Disease

**Fig. 2:**
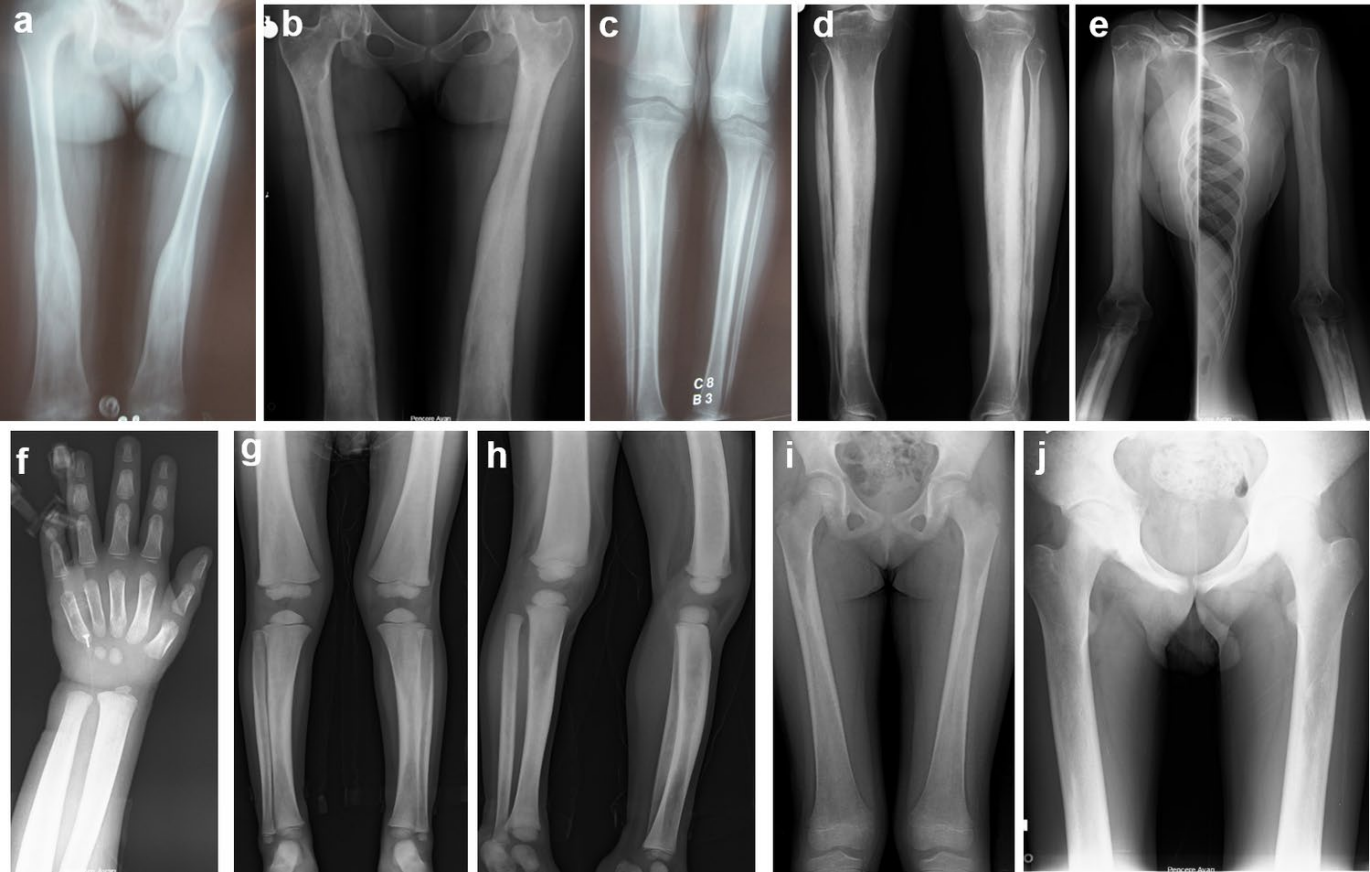
Patient 7 with Camurati–Engelmann disease at 9.5 (**a**, **c**) and 17 years (**b**, **d**, **e**). Note progressive diaphyseal involvement, periosteal hyperostosis, cortical thickening, and Erlermeyer flask deformity. Patient 12 at the age 3 (**f**–**h**) and her sister (P13) at 6.5 **(i)** and P11 at 18 years with Ghosal hematodiaphyseal dysplasia (**j**). Radiographs revealed diaphyseal widening and sclerosis, and cortical thickening

Four children (P7-P10) from unrelated families were diagnosed with Camurati–Engelmann disease, all presenting at the ages of between 6 and 15 years with easy fatigability, waddling gait, and limb pain, with symptom onset between 3 and 7 years. A monoallelic recurrent variant c.652C > T (p.Arg218Cys) in *TGFB1* was detected in all patients. At follow-up, three of them were found to have an asthenic habitus and their body mass index (BMI) SDS was − 8.71, − 3.55, and − 1.8. Radiologic findings on follow-up revealed progressive diaphyseal widening and sclerosis, Erlenmeyer flask deformity, and cortical thickening (Fig. 2a–e). In the two patients with BMD Z-values below − 2.5 and severe bone pain, pamidronate treatment was initiated, which resulted in significant pain relief during follow-up.

### Ghosal Hematodiaphyseal Dysplasia

Three patients (P11–P13) from two unrelated families diagnosed with Ghosal hematodiaphyseal dysplasia were found to have biallelic variants in *TBXAS1*, consisting of a known missense c.1235G > A (p.Arg412Gln) and a novel nonsense c.748G > T (p.Glu250*) variant. One patient presented at the age of 15.5 years with limb pain and knee joint restrictions that had persisted for five years, while another was admitted at the age of 3.3 years for severe anemia, thrombocytopenia, and hepatosplenomegaly, symptoms that had started at the age of one year. The third patient, a sibling of the second patient, was asymptomatic. Two patients suffering from bone pain and cytopenia underwent bone marrow aspiration, which revealed myelofibrosis. Radiographs showed enlarged diaphyses with diaphyseal sclerosis and thickened cortices in all patients at different ages (Fig. 2f–j). The patient with severe hematologic involvement responded well to treatment with prednisolone and symptoms resolved completely. No hematologic problems occurred in the other patients during follow-up.

### Juvenile Paget’s Disease Type 5

**Fig. 3:**
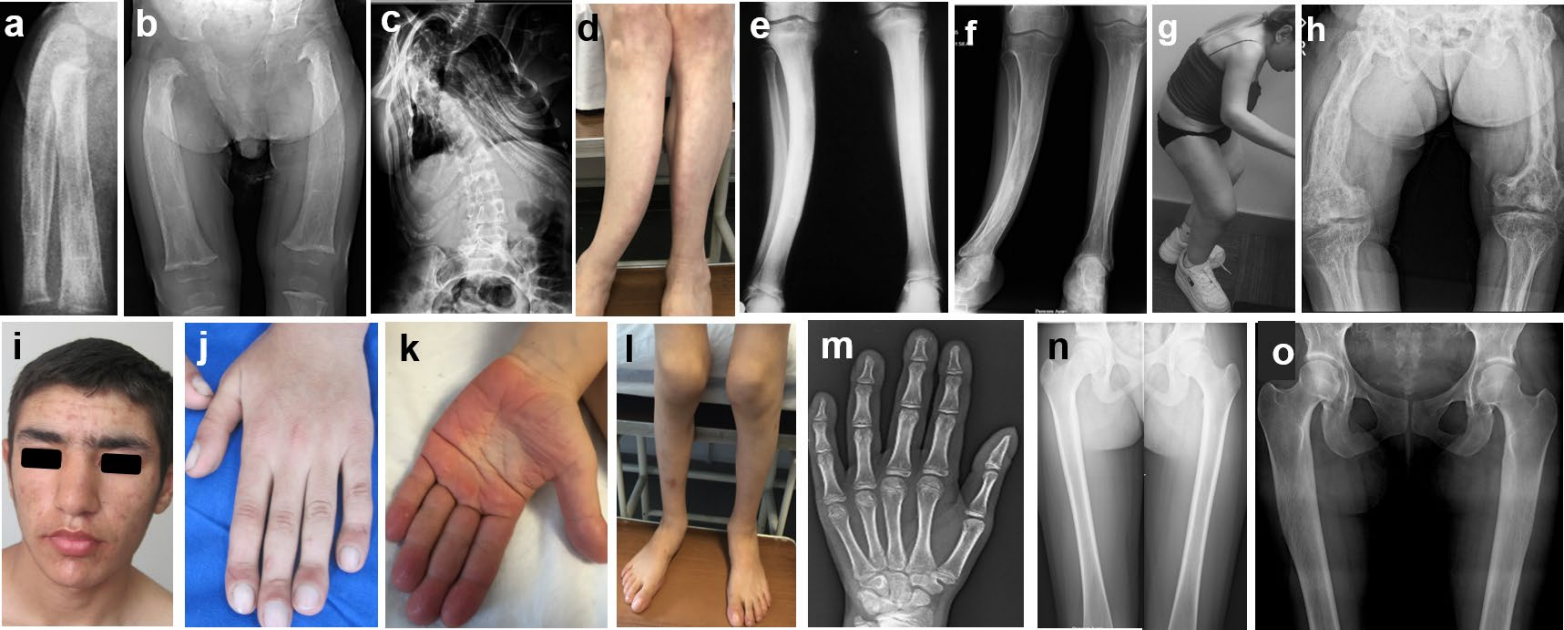
Photographs and radiographs of patients with Juvenile Paget’s disease-5 (**a**–**h**). Radiographs of P16 at 10 and 16 months of age, respectively (**a**, **b**) revealed osteopenia, multilayered, and irregular new bone formation at the diaphysis, and diaphyseal expansion. P14 showed severe scoliosis at the age of 23 years (**c**), anterior curvature of the tibia (**d**), and diffuse and patchy osteosclerosis of the long bones at the age of 13 and 23 years (**e**, **f**). P18 at the age of 30 years (**g**, **h**). Note the joint contractures with a forward bent posture with patchy osteosclerosis and coarse trabecular pattern. Photographs and radiographs of patients with hypertrophic osteoarthropathy type 1 (**i**–**o**). P24 at 17 years of age (**i**–**o**) showed coarse facial features (**i**), digital clubbing (**j**), enlargement and pachyderma of the hands (**k**), swelling of the knee (**i**–**l**). P22 at the age of 13 years (**m**, **n**), and P26 at the age of 24 years (**o**) showed acro-osteolysis (**m**) cortical thickening and periosteal hyperostosis (**n**, **o**)

Severe bone pain, fatigue, fractures, and skeletal deformities were observed in four children (P14, P16-P18) who had three different pathogenic variants in *TNFRSF11B*, including a previously reported missense variant c.193C > T (p.Cys65Arg), a novel nonsense c.814C > T (p.Gln272*), and a novel missense c.2 T > C (p.Met1Thr) variant. Fractures of the long bones, periosteal new bone formation, enlarged diaphyses, and osteopenia occurred in early childhood (Fig. 3a, b), while skeletal deformities such as severe scoliosis, kyphosis, and anterior curvature of the tibia as well as patchy osteosclerosis were observed in late childhood (Fig. 3c–e). Hearing loss was observed in early childhood in two of them. P14 and P18 were followed up to adulthood and showed severe short stature (− 5.8 SDS and − 7 SDS), early tooth loss and severe scoliosis or kyphosis, hip and knee joint contractures resulting in a forward leaning gait (Fig. 3g). However, P15, the affected sister of P14, had only generalized bone pain beginning in her early twenties, with radiographs showing osteopenia and a coarse trabecular pattern. With the exception of this patient, all patients had markedly elevated alkaline phosphatase levels, diaphyseal expansion, and a coarse trabecular bone pattern with patchy osteosclerosis (Fig. 3f,h).

### Primary Hypertrophic Osteoarthropathy

Nine patients (P19-P27), six males and three females, from five families were diagnosed with PHOAR-1 and three brothers (P28-P30) with PHOAR-2. Common symptoms included coarse facial features, digital clubbing with enlarged hands/feet, palmoplantar hyperkeratosis, and knee swelling (Fig. 3i–l). In addition, PHOAR-2 patients exhibited cutis gyrata. Radiographs showed enlarged diaphysis, cortical thickening, periosteal hyperostosis of the long bones, especially the femur, and acro-osteolysis (Fig. 3m–o). F13 (P19, 20) and F16 (P24, 25) had the c.310_311del (p.Leu104Alafs*3) variant in *HPGD.* We previously reported that the symptoms of P19 and P20 who were admitted in infancy with painful swelling and sweating of the hands and feet, cranial ossification defects, and enlarged diaphysis [17]. During follow-up, the enlargement of the diaphyses gradually improved, while the digital clubbing and swelling of the knees persisted, palmoplantar hyperkeratosis developed, and acro-osteolysis was observed on the radiographs of the hands. P21 with the c.175_176del (p.Leu59Valfs*8) variant was admitted at the age of 39 years and showed clubbing, palmoplantar hyperkeratosis, and hyperostosis of the long bones. In P22 and P23 with the c.418G > C (p.Ala140Pro) variant, symptoms appeared at the age of 6 and 12 years, with swelling of the knees, clubbing and palmoplantar hyperkeratosis. P26, carrying the same mutation, had only clubbing and hyperostosis of the long bones. The previously published clinical features of the three patients (P28-P30) with PHOAR-2 did not change during follow-up [16].

### Trichothiodystrophy Type 1

**Fig. 4:**
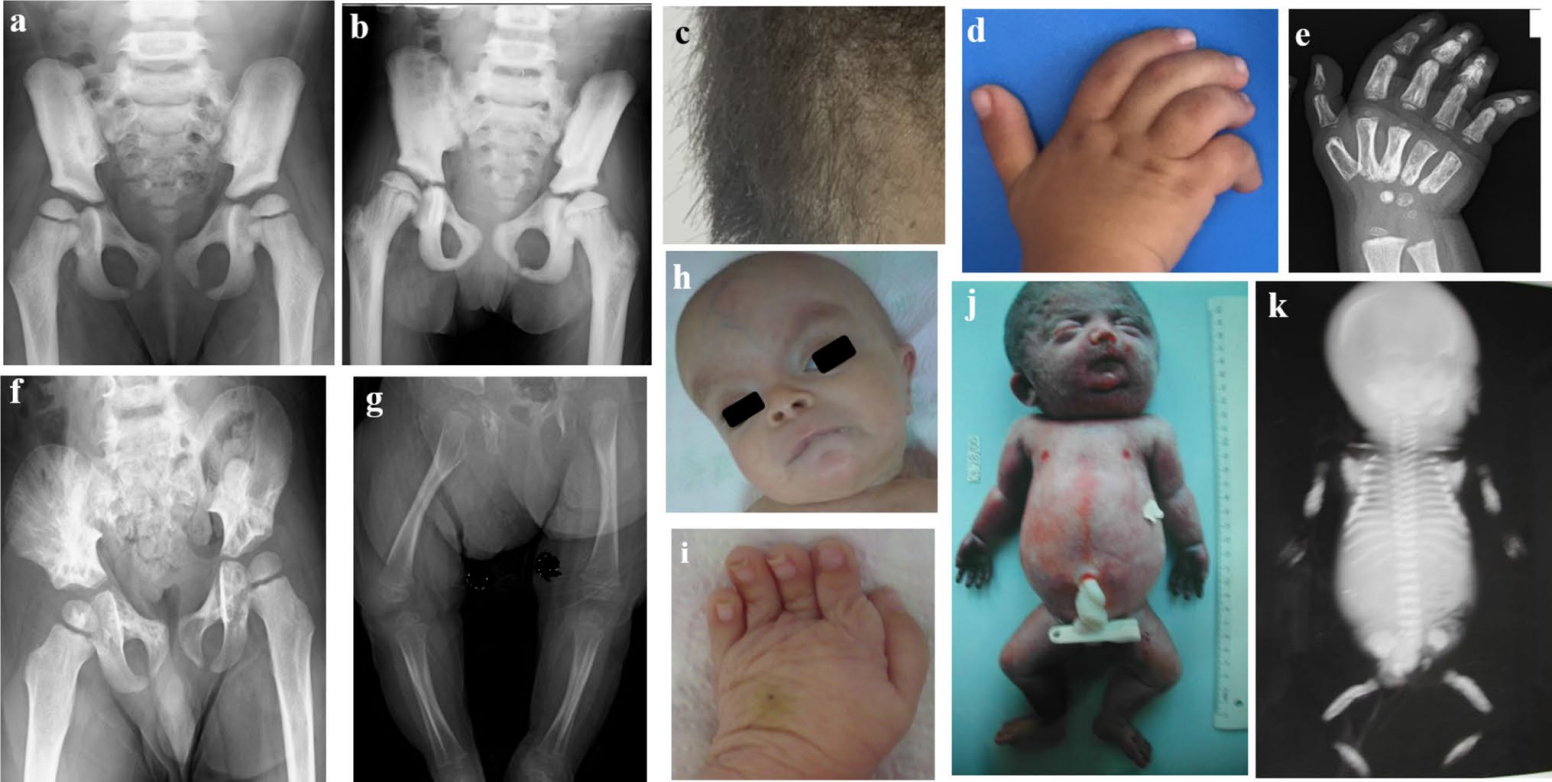
Patients with ultra-rare osteosclerotic disorders. P31 with Trichothiodystrophy-1 (**a**, **c**) at 3.8 years of age and 9.5 years of age (**b**). Note sparse, brittle hair, and diaphyseal and pelvic sclerosis, irregular acetabulum. P32 at 34 months of age with melorheostosis (**d**–**f**), right elbow contracture with dripping wax-like lesions and hyperostosis on pelvis radiograph. P33 with Lenz-Majewski hyperostotic dwarfism (**g**–**i**) at 6 months of age with prominent forehead, hypertelorism, severe brachydactyly with partial syndactyly, diaphyseal sclerosis, and metaphyseal osteopenia. A fetus with perinatal Caffey disease (**j**, **k**) showing generalized osteosclerosis and loss of cortex medulla border

Patient 31 was diagnosed with trichothiodystrophy that is characterized by ichthyosis, brittle hair, joint contractures, growth and developmental delay, and osteosclerosis. She was admitted at 3.8 years of age and followed for 5.3 years (Fig. 4a, b, c). Her height and head circumference SDSs were − 4.5 and − 3.6, respectively. Radiographs revealed osteosclerosis predominantly involving the cranium, spine, and pelvis. The BMD was markedly elevated (Z-score: + 4.9). During the follow-up period, at the age of 8.5 years, she developed severe hip dysfunction and difficulty walking. Molecular analysis identified compound heterozygous variants c. [2088G > T]; [423dup](p.[Trp696Cys]; [Leu59Valfs*8]) in *ERCC2*.

### Melorheosteosis

In P32 with melorheostosis, no pathogenic variant was found by exome sequencing. The patient had hemihypertrophy of the right lower limb, camptodactyly of the right hand (Fig. 4d), contracture of the right elbow, and hyperostosis with dripping wax-like lesions visible on radiographs (Fig. 4e).

### Lenz-Majewski Hyperostotic Dwarfism

Patient 33, diagnosed with Lenz-Majewski hyperostotic dwarfism, was a 6-month-old girl with developmental delay, feeding difficulties, hypotonia and facial dysmorphism including macrocephaly, flattened and broad nasal bridge, hypertelorism, anteverted nostrils, and micrognathia (Fig. 4f). Other features included sagging and wrinkled skin, brachydactyly, partial syndactyly, and rocker bottom feet (Fig. 4g). Radiologic examinations revealed short phalanges, increased bone density in the long bones (Fig. 4h), and hypoplasia of the 5th metatarsal.

### Caffey Disease

Caffey disease was diagnosed in a fetus with facial dysmorphism, low-set ears, long philtrum, thin lips, microretrognathia with a chin dimple, and a short neck on postmortem examination. The chest appeared narrow and the limbs were short (Fig. 4i). Radiographs revealed periosteal hyperostosis, irregular cortical densities, and a double-contoured diaphyseal cortical bones (Fig. 4j). Histopathologic analysis revealed thickening of the periosteum, subperiosteal mononuclear cell proliferation, and mesenchymal ossification abnormalities, confirming the diagnosis of prenatal cortical hyperostosis.

## Discussion

The prevalence of osteopetrosis and related osteoclastic disorders in Japan was investigated in a multicenter study involving 87 patients. It was found that 48.3% of the cohort suffered from osteopetrosis, 14.9% from pycnodysostosis and 36.8% from other sclerosing bone dysplasias [18]. In our single center study of 67 patients, 34% of this cohort had osteopetrosis and 15% had osteoclastic disorders. We have previously reported on a significant portion of this group [19]. Here we focus on osteosclerotic disorders in group 25, which accounted for 51% of our cohort [3].

Among the osteosclerotic diseases predominantly affecting the cranial bones, our cohort included four patients with craniometaphyseal dysplasia caused by monoallelic variants in *ANKH* and two patients with sclerosteosis-1 caused by a biallelic variant in *SOST*. Craniometaphyseal dysplasia is characterized by craniofacial dysmorphism and progressive sclerosis of the skull and metaphyseal widening. As the child grows, an Erlenmeyer flask deformity of the distal femur develops [20]. Three of the children with CMD presented here had marked facial dysmorphism and hearing loss, while one also had facial palsy. In early childhood, their radiographs showed diffuse cranial sclerosis, metaphyseal widening, radiolucent areas of the distal femoral metaphysis without involvement of the vertebrae and pelvis. The Erlenmeyer flask deformity developed over time. Complications of progressive cranial sclerosis, such as nasal obstruction, obstructive sleep apnea, and Chiari malformation requiring surgical correction, as seen in our patients, have been previously reported in CMD [21, 22]. Interestingly, two boys from the second family exhibited a severe phenotype, while their mildly affected mother remained asymptomatic into adulthood. Intrafamilial variability in CMD has already been reported [20]. Pathogenic variants in *ANKH* lead to both dominant-negative gain of function and loss of function in pyrophosphate transport [20]. Here, we reported a recurrent c.1124_1126del (p.Ser375del) variant in the first patient and a novel c.1145C > G (p.Thr382Arg) variant in the two siblings and their mother. The latter variant was classified as VUS, although it was predicted to be disease causing in the in silico databases and was not found in the gnomAD database.

Sclerosteosis-1 is an extremely rare disorder characterized by macrocephaly, overgrowth, midface hypoplasia, prognathism, syndactyly, hearing and vision loss, and markedly elevated BMD [23]. Similar to CMD, progressive sclerosis of the skull was observed on radiographs. In addition, sclerosis of the vertebrae and pelvis without metaphyseal enlargement was noted in sclerostosis. A biallelic nonsense variant c.372G > A (p.Trp124*) was identified in *SOST* in two siblings in our study who showed typical clinical features of sclerostosis. This variant is one of the well-documented pathogenic variants leading to loss of sclerostin function, resulting in unregulated WNT signaling and excessive bone formation [23].

In the group of osteosclerotic diseases with marked diaphyseal involvement, our cohort included four patients with Camurati-Engelmann disease and three patients with Ghosal hematodiaphyseal dysplasia. CED is characterized by progressive hyperostosis of the long bones, typically manifested by difficulty in walking, easy fatigability, bone pain and anemia [24, 25]. The recurrent c.652C > T (p.Arg218Cys) variant in *TGFB1* found in our four patients is a hotspot mutation previously identified in 60% of Camurati-Engelmann disease patients and is associated with increased TGFB1 activity [24]. Dysregulated TGF-β signaling leads to high bone turnover, which contributes to both osteosclerosis and decreased bone density [26]. The patients with CED had difficulty in walking that began between the ages of 3 and 7 years in our study. Their radiographs showed progressive enlargement of the diaphyses of the long bones with hyperostosis, Erlenmeyer flask deformity and narrowing of the medullary canal, which is consistent with previous reports [24]. Treatment with corticosteroids has been reported to be successful in reducing pain and weakness, improving gait, exercise tolerance, anemia and hepatosplenomegaly, and delaying bone hyperostosis [25, 27]. The use of bisphosphonates remains controversial [25]. However, our two patients with BMD below -2.5 received bisphosphonate therapy, which successfully relieved bone pain in both patients. Similar to these results, treatment with zoledronic acid was reported to reduce bone pain, decrease the size of hyperostotic lesions, improve weight gain, and improve mobility in two patients [28].

Ghosal hematodiaphyseal dysplasia is characterized by a waddling gait, diaphyseal dysplasia with cortical hyperostosis, corticosteroid-responsive anemia, and hypocellular bone marrow [12, 29]. *TBXAS1* encodes thromboxane synthase, a key enzyme in prostaglandin metabolism. Disruption of prostaglandin homeostasis in GHDD leads to a combination of skeletal abnormalities and hematologic manifestations. One of our patients, aged 15.5 years, presented with limb pain that had been present for five years, while the second patient with a novel nonsense variant had severe anemia that began at one year of age and responded well to steroid treatment. Radiographs of all patients showed diaphyseal sclerosis with thickened cortices and widened diaphyses. The third patient, the sibling of the second patient, was asymptomatic. In contrast, the other two patients, including a sibling of the severely affected patient, had no significant hematologic abnormalities at follow-up, highlighting the phenotypic heterogeneity of the disease. In view of the mild course in some cases, it was recommended that the family members of the affected individuals be examined [30].

Juvenile Paget’s disease-5 and primary hypertrophic osteoarthropathy are two osteosclerotic diseases with diaphyseal involvement that manifest in childhood. JPD-5 is a progressive disease characterized by short stature, hearing loss, skeletal deformities, increased fractures and very high serum alkaline phosphatase. Patients are categorized as having a severe phenotype due to the early onset of the disease with symptoms before the age of one year, hearing loss, severe short stature and progressive skeletal complications such as fractures, large joint contractures in infancy and long bone deformities and scoliosis in late childhood [31, 32]. In a collaborative study, we reported that a missense variant in the cysteine-rich ligand-binding domain was associated with a severe phenotype [15]. Similar to this patient, three of our patients from two families with a start codon missense variant and a nonsense variant also had a severe phenotype; severe short stature and skeletal deformities developed in adolescence and, consistent with the reported patients, osteopenia was observed in early childhood and, over time, osteosclerosis became apparent in two of them on skeletal radiographs [33]. However, the sister of the patient with the nonsense mutation had a mild course at the age of 22 years, characterized by generalized bone pain without bone deformities, highlighting the intrafamilial phenotypic variability. In contrast to other osteosclerotic diseases, the rapid bone turnover that occurs in the pathogenesis of this group results in discomfort and deformities associated with osteopenia. We have already reported that the skeletal findings in one of the patients improved significantly after treatment with calcitonin in infancy [34]. In this patient and our other patient, who was followed up until the age of 37 years, a coarse trabecular bone pattern with patchy osteosclerosis was present, but severe bone pain and osteopenia were evident. Treatment with bisphosphonates resulted in effective pain relief when used intermittently according to established treatment approaches [31]. Although the use of denosumab in JPD-5 leads to normalization of alkaline phosphatase levels and improvement of bone pain, it is not recommended due to the development of severe hypocalcemia [35].

Primary hypertrophic osteoarthropathy types 1 and 2 are characterized by the clinical triad of digital clubbing, arthropathy and pachydermoperiostitis. Patients with PHOAR-2 also have a prominent forehead furrow, prominent facial wrinkles, ptosis and thickened eyelids that appeared at puberty [13, 16]. In our PHOAR cohort, the mean age at admission of PHOAR-1 patients (12.7 years) is lower than that of PHOAR-2 patients (19 years), which is consistent with the typical onset of PHOAR-1 in childhood and PHOAR-2 in adolescence [13]. To date, a total of 89 patients with PHOAR-1 have been reported [13]. In PHOAR-1 patients we previously reported, diaphyseal enlargement gradually regressed with age, while digital clubbing and knee swelling persisted and palmoplantar hyperkeratosis developed. Radiographs showed periosteal thickening of the long bones, especially the femur, and acro-osteolysis on the hands [17]. In six of these patients who reached adulthood, the classic findings were observed: Digital clubbing, periosteal thickening of long bones, and acro-osteolysis on radiographs. Some patients had palmoplantar hyperkeratosis or a coarse facial and skin appearance. After our publication of these patients, the c.310_311delCT (p.Leu104Alafs*3) mutation was identified as a recurrent variant in *HPGD* and recognized as a hotspot mutation within the Asian ethnic group [36]. It has been suggested that non-steroidal anti-inflammatory drugs can inhibit COX and reduce prostaglandin levels, making them a suitable treatment option for PHOAR-1, leading to symptomatic relief of painful periostosis and arthritis [13]. However, in the studies conducted, including our study, no beneficial effect on recurrent attacks of painful swelling and sweating of the hands and feet or on radiologic findings was observed. The cutis gyrata deepens with age in PHOAR-2 patients. Cosmetic procedures on the severe cutis gyrata, on the forehead of P27, were not helpful.

Trichothiodystrophy-1 with axial osteosclerosis is a very rare disease related to nucleotide excision repair and associated with biallelic variants in *ERCC2*. It is characterized by sclerotic changes of axial bones, brittle hair and photosensitivity [3, 6]. We reported a patient who was diagnosed with central osteosclerosis associated with trichothiodystrophy-1. During follow-up, the patient developed significant difficulty walking and severe hip joint pain at the age of 8.5 years, similar to the previously reported patient [37]. We clearly observed joint stiffness and also sclerosis of the skull, vertebrae and pelvis, which is the characteristic feature of the disease progressing with age, similar to the few patients reported [38].

Lenz-Majewski hyperostotic dwarfism showed typical clinical findings in one of our patients, including severe prenatal short stature, a dismorphic face, and radiologic features such as short or absent metacarpals and phalanges, diaphyseal thickening, and metaphyseal hypostosis [5, 39]. To date, only twenty patients have been documented, 12 of whom received molecular confirmation [40].

Heterozygosity for a *COL1A1* mutation was found in the lung tissue of a fetus with a severe form of prenatal cortical hyperostosis [41]. In our cohort, one fetus was diagnosed with perinatal Caffey disease with typical clinical, radiologic, and histopathologic findings.

Melorheostosis often presents with a classic “dripping candle wax” radiographic appearance. Despite no pathogenic variant detected through exome sequencing, recent studies link melorheostosis to somatic mutations, particularly in the *MAP2K1*, which plays a crucial role in the RAS-MAPK signaling pathway, implicated in bone remodeling and sclerosis [9]. Our patient, in whom we could not detect mutations in the blood, had unilateral camptodactyly and hyperostosis, which are typical features of the syndrome, as well as dripping waxy lesions on radiographs. The absence of a detectable variant in blood DNA is not unexpected in melorheostosis, as somatic mutations are typically restricted to the affected tissue. This highlights the mosaic nature of the condition and indicates that genetic testing of affected tissue may be required to identify the underlying mutation.

## Conclusion

Primary hypertrophic osteoarthropathy was the most common, accounting for 35% of 34 the patients with osteosclerotic diseases. JPD-5 and CED patients showed osteopenia in early childhood, while osteosclerosis developed in late childhood. The clinical findings in PHOAR-1 improved with age, whereas they were progressive in trichothiodystrophy-1 and JPD-5. Bone marrow involvement was developed in some patients with CED and GHDD. PHOAR was characterized by clubbing and pachydermia, trichothiodystrophy-1 by ectodermal findings, and Lenz-Majewski hyperostotic dwarfism by dysmorphic facies and short fingers.Clinical variability within families and among individuals with the same mutation was observed in patients with CED, CMD, GHDD, and JPD-5. Patients with GHDD and bone marrow involvement showed significant improvement with cortisone treatment, while those with JPD-5 and CED experiencing osteopenia responded well to calcitonin or bisphosphonates. Effective treatments at the molecular level for osteosclerotic disorders are still at the experimental stage. Our study reporting the basic characteristics and long-term follow-up findings of different types of osteosclerotic disorders may contribute to a deeper understanding of bone biology and potential treatment strategies.

## Supplementary Information

Below is the link to the electronic supplementary material.

Supplementary file1 (DOCX 28 KB)

Supplementary file2 (DOCX 31 KB)

## References

[1] Boudin E, Van Hul W (2018) Sclerosing bone dysplasias. Best Pract Res Clin Endocrinol Metab 32(5):707–723. 10.1016/j.beem.2018.06.003 30449550 10.1016/j.beem.2018.06.003

[2] De Ridder R, Boudin E, Mortier G, Van Hul W (2018) Human genetics of sclerosing bone disorders. Curr Osteoporos Rep 16(3):256–268. 10.1007/s11914-018-0439-7 29656376 10.1007/s11914-018-0439-7

[3] Unger S, Ferreira CR, Mortier GR et al (2023) Nosology of genetic skeletal disorders: 2023 revision. Am J Med Genet A 191(5):1164–1209. 10.1002/ajmg.a.63132 36779427 10.1002/ajmg.a.63132 PMC10081954

[4] Guerin A, Dupuis L, Mendoza-Londono R (2012) Caffey disease. In: Adam MP, Feldman J, Mirzaa GM, Pagon RA, Wallace SE, Amemiya A (Eds). GeneReviews®. Seattle 22855962

[5] Sousa SB, Jenkins D, Chanudet E et al (2014) Gain-of-function mutations in the phosphatidylserine synthase 1 (PTDSS1) gene cause Lenz-Majewski syndrome. Nat Genet 46(1):70–76. 10.1038/ng.2829 24241535 10.1038/ng.2829

[6] Harreld JH, Smith EC, Prose NS, Puri PK, Barboriak DP (2010) Trichothiodystrophy with dysmyelination and central osteosclerosis. AJNR Am J Neuroradiol 31(1):129–130. 10.3174/ajnr.A1665 20075106 10.3174/ajnr.A1665 PMC7964056

[7] Huybrechts Y, Mortier G, Boudin E, Van Hul W (2020) WNT signaling and bone: lessons from skeletal dysplasias and disorders. Front Endocrinol (Lausanne) 11:165. 10.3389/fendo.2020.00165 32328030 10.3389/fendo.2020.00165 PMC7160326

[8] Tang Y, Wu X, Lei W et al (2009) TGF-beta1-induced migration of bone mesenchymal stem cells couples bone resorption with formation. Nat Med 15(7):757–765. 10.1038/nm.1979 19584867 10.1038/nm.1979 PMC2727637

[9] Wordsworth P, Chan M (2019) Melorheostosis and osteopoikilosis: a review of clinical features and pathogenesis. Calcif Tissue Int 104(5):530–543. 10.1007/s00223-019-00543-y 30989250 10.1007/s00223-019-00543-y

[10] Whyte MP (2006) Paget’s disease of bone and genetic disorders of RANKL/OPG/RANK/NF-kappaB signaling. Ann N Y Acad Sci 1068:143–164. 10.1196/annals.1346.016 16831914 10.1196/annals.1346.016

[11] Daroszewska A, Ralston SH (2006) Mechanisms of disease: genetics of Paget’s disease of bone and related disorders. Nat Clin Pract Rheumatol 2(5):270–277. 10.1038/ncprheum0172 16932700 10.1038/ncprheum0172

[12] Geneviève D, Proulle V, Isidor B et al (2008) Thromboxane synthase mutations in an increased bone density disorder (Ghosal syndrome). Nat Genet 40(3):284–286. 10.1038/ng.2007.66 18264100 10.1038/ng.2007.66

[13] Lu Q, Xu Y, Zhang Z, Li S, Zhang Z (2023) Primary hypertrophic osteoarthropathy: genetics, clinical features and management. Front Endocrinol (Lausanne) 14:1235040. 10.3389/fendo.2023.1235040 37705574 10.3389/fendo.2023.1235040 PMC10497106

[14] Williams CJ (2016) The role of ANKH in pathologic mineralization of cartilage. Curr Opin Rheumatol 28(2):145–151. 10.1097/BOR.0000000000000247 26599446 10.1097/BOR.0000000000000247

[15] Chong B, Hegde M, Fawkner M et al (2003) Idiopathic hyperphosphatasia and TNFRSF11B mutations: relationships between phenotype and genotype. J Bone Miner Res 18(12):2095–2104. 10.1359/jbmr.2003.18.12.2095 14672344 10.1359/jbmr.2003.18.12.2095

[16] Seifert W, Kühnisch J, Tüysüz B, Specker C, Brouwers A, Horn D (2012) Mutations in the prostaglandin transporter encoding gene SLCO2A1 cause primary hypertrophic osteoarthropathy and isolated digital clubbing. Hum Mutat 33(4):660–664. 10.1002/humu.22042 22331663 10.1002/humu.22042

[17] Tüysüz B, Yılmaz S, Kasapçopur Ö et al (2014) Primary hypertrophic osteoarthropathy caused by homozygous deletion in HPGD gene in a family: changing clinical and radiological findings with long-term follow-up. Rheumatol Int 34(11):1539–1544. 10.1007/s00296-014-3037-8 24816859 10.1007/s00296-014-3037-8

[18] Sawamura K, Mishima K, Matsushita M, Kamiya Y, Hm K (2022) A cross-sectional nationwide survey of osteosclerotic skeletal dysplasias in Japan. J Orthop Sci 27(5):1139–1142. 10.1016/j.jos.2021.05.012 34275722 10.1016/j.jos.2021.05.012

[19] Tüysüz B, Usluer E, Uludağ Alkaya D et al (2023) The molecular spectrum of Turkish osteopetrosis and related osteoclast disorders with natural history, including a candidate gene, CCDC120. Bone 177:116897. 10.1016/j.bone.2023.116897 37704070 10.1016/j.bone.2023.116897

[20] Reichenberger E, Chen IP (2007) Craniometaphyseal dysplasia, autosomal dominant. In: Adam MP, Feldman J, Mirzaa GM, Pagon RA, Wallace SE, Amemiya A (Eds). GeneReviews®. Seattle 20301634

[21] Mintz S, Velez I (2004) Craniometaphyseal dysplasia associated with obstructive sleep apnoea syndrome. Dentomaxillofac Radiol 33(4):262–266. 10.1259/dmfr/17660567 15533982 10.1259/dmfr/17660567

[22] Tanaka M, Arataki S, Sugimoto Y, Takigawa T, Tetsunaga T, Ozaki T (2013) Chiari type I malformation caused by craniometaphyseal dysplasia. Acta Med Okayama 67(6):385–389. 10.18926/AMO/52012 24356723 10.18926/AMO/52012

[23] Appelman-Dijkstra N, Van Lierop A, Papapoulos S (2002) SOST-related sclerosing bone dysplasias. In: Adam MP, Feldman J, Mirzaa GM, Pagon RA, Wallace SE, Amemiya A (Eds). GeneReviews®. Seattle 36508511

[24] Janssens K, Vanhoenacker F, Bonduelle M et al (2006) Camurati-Engelmann disease: review of the clinical, radiological, and molecular data of 24 families and implications for diagnosis and treatment. J Med Genet 43(1):1–11. 10.1136/jmg.2005.033522 15894597 10.1136/jmg.2005.033522 PMC2564495

[25] Wallace SE, Wilcox WR (2004). Camurati-Engelmann disease. In: Adam MP, Feldman J, Mirzaa GM, Pagon RA, Wallace SE, Amemiya A (Eds). GeneReviews®. Seattle 20301335

[26] Van Hul W, Boudin E, Vanhoenacker FM, Mortier G (2019) Camurati-Engelmann disease. Calcif Tissue Int 104(5):554–560. 10.1007/s00223-019-00532-1 30721323 10.1007/s00223-019-00532-1

[27] Mwasamwaja AO, Mkwizu EW, Shao ER, Kalambo CF, Lyaruu I (2018) Hamel BC (2018) Camurati-Engelmann disease: a case report from sub-Saharan Africa. Oxf Med Case Rep 7:036. 10.1093/omcr/omy036 10.1093/omcr/omy036 PMC6049015 30034812

[28] Baroncelli GI, Ferretti E, Pini CM, Toschi B, Consolini R, Bertelloni S (2017) Significant improvement of clinical symptoms, bone lesions, and bone turnover after long-term zoledronic acid treatment in patients with a severe form of Camurati-Engelmann disease. Mol Syndromol 8(6):294–302. 10.1159/000479859 29230158 10.1159/000479859 PMC5701277

[29] Joy P, Yoganathan S, Korula S et al (2021) Ghosal hematodiaphyseal dysplasia and response to corticosteroid therapy. Am J Med Genet A 185(2):596–599. 10.1002/ajmg.a.61961 33185009 10.1002/ajmg.a.61961

[30] Mazaheri P, Nadkarni G, Lowe E et al (2010) Ghosal hematodiaphyseal dysplasia: a rare cause of a myelophthisic anemia. Pediatr Blood Cancer 55(6):1187–1190. 10.1002/pbc.22662 20672367 10.1002/pbc.22662

[31] Polyzos SA, Cundy T, Mantzoros CS (2018) Juvenile Paget disease. Metabolism 80:15–26. 10.1016/j.metabol.2017.10.007 29080812 10.1016/j.metabol.2017.10.007

[32] Grasemann C, Unger N, Hövel M et al (2017) Loss of functional osteoprotegerin: more than a skeletal problem. J Clin Endocrinol Metab 102(1):210–219. 10.1210/jc.2016-2905 27809640 10.1210/jc.2016-2905

[33] Ralston SH, Taylor JP (2019) Rare ınherited forms of paget’s disease and related syndromes. Calcif Tissue Int 104(5):501–516. 10.1007/s00223-019-00520-5 30756140 10.1007/s00223-019-00520-5 PMC6779132

[34] Tüysüz B, Mercimek S, Ungür S, Deniz M (1999) Calcitonin treatment in osteoectasia with hyperphosphatasia (juvenile Paget’s disease): radiographic changes after treatment. Pediatr Radiol 29(11):838–841. 10.1007/s002470050708 10552064 10.1007/s002470050708

[35] Vanderniet JA, Vivian Szymczuk V, Högler W et al (2024) Management of RANKL-mediated disorders with Denosumab in children and adolescents: a global expert guidance document. J Clin Endocrinol Metab 109:1371–1382. 10.1210/clinem/dgad657 38041865 10.1210/clinem/dgad657 PMC11031248

[36] Chen Y, Li G, Xu Y et al (2018) Targeted exome sequencing identified a novel mutation hotspot and a deletion in Chinese primary hypertrophic osteoarthropathy patients. Clin Chim Acta 487:264–269. 10.1016/j.cca.2018.10.005 30292630 10.1016/j.cca.2018.10.005

[37] DiGiovanna JJ, Randall G, Edelman A et al (2022) Debilitating hip degeneration in trichothiodystrophy: association with ERCC2/XPD mutations, osteosclerosis, osteopenia, coxa valga, contractures, and osteonecrosis. Am J Med Genet A 188(12):3448–3462. 10.1002/ajmg.a.62962 36103153 10.1002/ajmg.a.62962 PMC9669218

[38] Wakeling EL, Cruwys M, Suri M, Brady AF, Aylett SE (2004) Hall C (2004) Central osteosclerosis with trichothiodystrophy. Pediatr Radiol 34(7):541–546. 10.1007/s00247-004-1207- 15148554 10.1007/s00247-004-1207-7

[39] Whyte MP, Blythe A, McAlister WH, Nenninger AR, Bijanki VN, Mumm S (2015) Lenz-Majewski hyperostotic dwarfism with hyperphosphoserinuria from a novel mutation in PTDSS1 encoding phosphatidylserine synthase 1. J Bone Miner Res 30(4):606–614. 10.1002/jbmr.2398 25363158 10.1002/jbmr.2398

[40] Maden Bedel F, Balasar Ö, Erol Aytekin S, Keleş S, Çaksen H (2024) Lenz-Majewski syndrome and recurrent otitis media: are they related or not? Eur J Med Genet 68:104910. 10.1016/j.ejmg.2024.104910 38262577 10.1016/j.ejmg.2024.104910

[41] Kamoun-Goldrat A, Martinovic J, Saada J et al (2008) Prenatal cortical hyperostosis with COL1A1 gene mutation. Am J Med Genet A 146A(14):1820–1824. 10.1002/ajmg.a.32351 18553566 10.1002/ajmg.a.32351

