# Supplementary material for: Insights into Natural History, Phenotypic, and Molecular Spectrum in a Large Cohort of Osteosclerotic Disorders
Source: Calcif Tissue Int. 2025 Apr 8;116(1):59. doi: 10.1007/s00223-025-01366-w (PMC11978542; doi:10.1007/s00223-025-01366-w)
Supplement: Supplementary file 1 — Supplementary file1 (DOCX 28 KB) [file 223_2025_1366_MOESM1_ESM.docx]

**Table S1** Comparison of the clinical characteristics of the patients according to the different phenotypes

|  | **Craniometaphyseal dysplasia (n:4)** | **Sclerosteosis-1 (n:2)** | **Camurati-Engelmann disease (n:4)** | **Ghosal hematodiaphyseal dysplasia (n:3)** | **Juvenile Paget disease-5 (n:5)** | **Primary hypertrophic osteoarthropathy (n:12)** | **Trichothiodystroph (n:1)** | **Melorheosteosis (n:1)** | **Lenz- Majewski hyperostotic dwarfism(n:1)** | **Prenatal Caffey disease (n:1)** |
| --- | --- | --- | --- | --- | --- | --- | --- | --- | --- | --- |
| **Admission age (median, years)** | 4.6  (1.4-29) | 40.5  (37-44) | 9.7  (6- 14) | 7.3  (3.3-15.5) | 0.9  (0.41- 12.9) | 14.8  (0.7-39) | 3.8 | 0.7 | 0.5 | Fetus |
| **Follow-up duration (median, years)** | 18.1  (11.9-22) | 4.7  (1-7) | 5.8  (1.1-10.6) | 5.2  (1-6.2) | 6.6  (2-21.9) | 3  (1.6-8.3) | 5.3 | 9.8 | - | - |
| **Clinical features** | | | | | | | | | | |
| **Facial features** | All had long face,broad forehead: hypertelorism,midface hypoplasia, prognathism | Both had macrocephaly, long face, broad forehead, hypertelorism, midface hypoplasia, prognathism | - | - | - | - | - | - | Broad forehead, hypertelorism,  antevert nostrils,micrognathia | Low-set ears, long philtrum, thin lips, microretrognathia,  short neck |
| **Short stature** | - | - | - | - | 2/5 | - | 1/1 | - | 1/1 | - |
| **Cranial nerve involvement** | Facial palsy:1/4  Hearing loss: 4/4  Swallowing dysfunction:1/4 | Facial palsy:2/2  Hearing loss: 2/2  Optic atrophy:1/2 | - | - | Hearing loss: 2/5 | - | - | - | - | - |
| **Fractures** | 1/4 | - | - | 1/4 | 4/5 | - | - | - | - | - |
| **Other** | Obstructive sleep apnea:1/4  Chiari malformation and cerebellar herniation:1/4 | Tall stature:1/2  Partial syndactyly:2/2 | Asthenic habitus:3/4  Hypermobility: 2/4  Walking difficulty: 4/4  Pain: 3/4  Anemia:1/4  Delayed puberty:1/4 | Bicytopenia:1/3  Myelofibrosis:2/3 | Joint contractures: 2/5  Early tooth loss:2/5  Markedly elevated ALP:4/5 | Enlarged hand and feet:9/12  Clubbing:11/12  Swelling of large joints:10/12  Cutis gyrate:2/12  Palmoplantar hyperkeratosis:11/12 | Ichthyosis  Joint contractures  Developmental delay | Right hemihypertrophy,  right hand camptodactly,  right elbow contracture | Sagging and wrinkled skin, brachydactyly, partial syndactyly, rocker bottom feet | Polyhydramniosis |
| **Radíological features** | | | | | | | | | | |
| **Marked involvement of osteosclerosis** | Severe sclerosis of  cranial bones, and paranasal sinuses  Diaphyseal sclerosis of long bones in early childhood | Severe sclerosis of cranial bones, paranasal sinuses, long bones, vertebra, pelvis, clavicles, ribs, hands | Progressive sclerosis of long bone diaphysis | Mild to moderate sclerosis of long bone diaphysis | Patchy sclerosis and coarse trabecular pattern of long bone diaphysis | Periosteal hyperostosis | Moderate sclerosis of cranium, vertebra, pelvis, and clavicles | Dripping wax-like sclerosis on pelvis, right metacarpals and phalanges, bilateral metatarsals, epiphysis of bilateral proximal tibia, proximal and distal femur, proximal humerus | Moderate sclerosis of long bones diaphysis | Periosteal hyperostosis  of ribs and long bones |
| **Other radiological findings** | Widening and radiolucency of the metaphysis (early childhood)  Erlenmeyer flask deformity (late childhood) | Cortical thickening  Scoliosis | Progressive diaphyseal widening  Periosteal hyperostosis  Cortical thickening  Erlenmeyer flask deformity | Diaphyseal widening  Cortical thickening | Osteopenia in early childhood  Diaphyseal widening  Severe scoliosis Anterior curvature of tibia | Cranial ossification defect with wormian bones  Diaphyseal widening  Acro-osteolysis  Cortical thickening | Coxa valga | - | Short phalanges, hypoplasia of 5^th^ metacarpal and metatarsal bones |  |
| **DEXA Z score**  < -2 (n:)  > +2 (n:) | > +2 (n:1) | >+2 (n:1) | < -2 (n:2)  > +2 (n:1) | - | < -2 (n:3) | - | > +2 | - | NA | NA |
| **Treatment/surgery** | | | | | | | | | | |
|  | Surgery for nasal obstruction 3/4  Chiari malformation/ cerebellar herniation surgery: 1/4 | - | Pamidronate:2/4 | Prednisolone:1/4 | Pamidronate/teriparatide//calcitonine:4/5 | - | - | - | - | - |

ALP: Alkaline phosphatase; DEXA: dual-energy X-ray absorptiometry; NA: not available
