# Supplementary material for: Insights into Natural History, Phenotypic, and Molecular Spectrum in a Large Cohort of Osteosclerotic Disorders
Source: Calcif Tissue Int. 2025 Apr 8;116(1):59. doi: 10.1007/s00223-025-01366-w (PMC11978542; doi:10.1007/s00223-025-01366-w)
Supplement: Supplementary file 2 — Supplementary file2 (DOCX 31 KB) [file 223_2025_1366_MOESM2_ESM.docx]

**Table S2: Clinical and Radiological Features of the Patients with Osteosclerotic Disorders**

| **Family Number** | **1** | **2** | | | **3** | | **4** | **5** | **6** | **7** |
| --- | --- | --- | --- | --- | --- | --- | --- | --- | --- | --- |
| **Patient Number** | **1** | **2** | **3** | **4** | **5** | **6** | **7** | **8** | **9** | **10** |
| **Diagnosis** | **Craniometaphyseal dysplasia** | | | | **Sclerosteosis 1** | | **Camurati-Engelmann disease** | | | |
| **Gender(F/M)** | M | M | M | F | F | M | F | F | F | M |
| **Parental**  **Consanguinity** | - | - | | - | - | | - | - | - | - |
| **Age at admission(years)** | 1.4 | 3.8 | 5.5 | 29 | 37 | 44 | 9.7 | 14.7 | 7.3 | 6.7 |
| **Age at last examination** | 16.3 | 25.8 | 19.7 | 51 | 44 | 45 | 20.3 | 15.8 | 7.4 | 7 |
| **Height SDS at admission** | 0.08 | -1.66 | +0.78 | -0.1 | 1 | 2.4 | -1.41 | -1.75 | -1.56 | -1.86 |
| **Height SDS at last examination** | 1.01 | -0.84 | +0.78 | -0.1 | 1 | 2.4 | -1.19 | -0.73 | NA | NA |
| **Clinical findings** |  |  |  |  |  |  |  |  |  |  |
| **Admission symptom** | Facial paralysis | Facial dysmorphism | Facial dysmorphism | No symptom | Facial dysmorphism | Facial dysmorphism | Limb pain, walking difficulty | Limb pain, walking difficulty | Walking difficulty | Walking difficulty, limb pain |
| **Initial clinical findings** | Midface hypoplasia, hypertelorism, broad nasal root,  Facial paralysis  Nasal obstruction  Chronic dacryocystitis | Midface hypoplasia, hypertelorism, broad nasal root, Hearing loss | Midface hypoplasia, hypertelorism, broad nasal root, Hearing loss | Midface hypoplasia, prognathism long face, Hearing loss | long face  Macrocephaly long face, broad forehead, midface hypoplasia, broad nasal root, prognathism  Facial paralysis  Vision loss  Hearing loss | Macrocephaly long face, broad forehead, midface hypoplasia, broad nasal root, prognathism  Facial paralysis  Hearing loss | Blue sclera  Hypermobility  Asthenic habitus | Hypermobility  Asthenic habitus | - | Asthenic habitus |
| **Additional follow-up clinical findings** | long face, prognathism  Recurrent facial paralysis  OSAS (8.5 years) | long face, - prognathism | Swallowing dysfunction due to Chiari malformation  Cerebellar herniation | - | - | - | Pain resolved Irregular menstrual cycles | Delayed puberty | - | - |
| **Hematological involvement** | - | - | - | - | - | - | - | - | Anemia | - |
| **Eye problems** | Optic disc pallor ( 7 years) | - | - | - | Optic atrophy, strabismus | - | Exophthalmos | - | - | Myopia |
| **Hearing loss** | + (3.5 years) | + (3.8 years) | + (5 years) | + (40 years) | + (5 years) | + (5 years) | - | - | - | - |
| **Renal anomaly** | - | - | - | - | Right duplicated collecting system | - | - | - | - | - |
| **Cardiac anomaly** | - | - | - | - | Mild aortic regurgitation | - | - | - | - | - |
| **Intellectual disability** | - | - | - | - | Mild | Mild | - | - | - | - |
| **Fracture history (n) (age)** | + (1)  (10.5 years) | - | - | -- | - | - | - | - | - | - |
| **Other** | - | - | - | - | Myasthenia gravis, epilepsy | - | - | Developmental hip dysplasia, Celiac disease | - | - |
| **Radiological findings** |  |  |  |  |  |  |  |  |  |  |
| **Initial radiological findings** | Severe sclerosis of  cranial bones, and paranasal sinuses  Widening and radiolucency of the metaphysis and diaphyseal sclerosis  of the long bones | Severe sclerosis of  cranial bones, and paranasal sinuses  Widening of metaphysis | Severe sclerosis of cranial bones, and paranasal sinuses  Widening and radiolucency of the metaphysis and diaphyseal sclerosis  of the long bones | Severe sclerosis of cranial bones, and paranasal sinuses  Erlenmeyer flask deformity | Severe sclerosis of  cranial bones, and paranasal sinuses, long bones, vertebra, pelvis, clavicles, ribs, hand  Cortical thickening  Mild thoracic scoliosis | Severe sclerosis of  cranial bones, and paranasal sinuses, long bones, vertebra, pelvis, clavicles, ribs, hand  Cortical thickening  Mild lomber scoliosis | Mild to moderate sclerosis of long bone diaphysis  Erlenmeyer flask deformity  Periosteal hyperostosis  Cortical thickening | Moderate sclerosis of long bone diaphysis  Erlenmeyer flask deformity  Periosteal hyperostosis  Cortical thickening  Medullary canal narrowed  Coxa valga  Thoracic scoliosis | Mild sclerosis of long bone diaphysis  Erlenmeyer flask deformity  Periosteal hyperostosis  Cortical thickening | Moderate sclerosis of long bone diaphysis  Periosteal hyperostosis  Cortical thickening  Medullary canal narrowed |
| **Additional follow-up radiological findings** | Erlenmeyer flask deformity  Radiolucency of the metaphysis of the long bones and diaphyseal sclerosis resolved | Erlenmeyer flask deformity | Erlenmeyer flask deformity  Radiolucency of the metaphysis of the long bones and diaphyseal sclerosis resolved | Mild thoracic scoliosis developed | - | - | Diaphyseal sclerosis, cortical thickening progressed  Medullary canal narrowed | - | - | - |
| **DEXA (initial)** | +2.15 | NA | NA | NA | +15.1 | NA | -3.0 | -5.0 | -1.2 | +5.1 |
| **DEXA (last)** | +0.5 | +1.8 | +1.6 | NA | NA | NA | -1.8 | -4.8 | NA | NA |
| **Treatment and surgery history** | Recurrent surgeries for nasal obstruction  Orthodontic treatment | Recurrent surgeries for nasal obstruction | Surgery for nasal obstruction (12 years) and Chiari malformation  (17 years) | - | - | - | Pamidronate | Pamidronate | - | - |

**Table S2: Continuous**

| **Family Number** | **8** | **9** | | **10** | | **11** | | **12** | **13** | |
| --- | --- | --- | --- | --- | --- | --- | --- | --- | --- | --- |
| **Patient Number** | **11** | **12** | **13** | **14** | **15** | **16** | **17** | **18** | **19** | **20** |
| **Diagnosis** | **Ghosal hematodiaphyseal dysplasia** | | | **Juvenile Paget Disease** | | | | | **Primary hypertrophic osteoarthropathy-1** | |
| **Gender(F/M)** | M | M | F | F | F | M | M | F | M | M |
| **Parental**  **Consanguinity** | - | + | | + | | + | | + | + | |
| **Age at admission(years)** | 15.5 | 3.3 | 6.6 | 12.9 | 22 | 0.8 | 0.4 | 1.3 | 0.7 | 0.3 |
| **Age at last examination** | 20.7 | 9.5 | 7.6 | 37 | 24 | 1.3 | 0.4 | 23 | 9 | 2.3 |
| **Height SDS at admission** | 1.17 | -0.33 | 0.27 | -3.19 | -1.72 | -2.32 | -1.2 | -1.8 | -1.5 | -1.7 |
| **Height SDS at last examination** | 1.59 | -1.69 | 0.5 | -5.8 | -1.72 | -1.87 | -1.2 | -7.0 | NA | NA |
| **Clinical findings** |  |  |  |  |  |  |  |  |  |  |
| **Admission symptom** | Limited knee joint movement, pain | Severe anemia, myelofibrosis | No symptom | Cataract, hearing loss, fracture, walking difficulty | Bone pain | Fracture | Fracture | Walking difficulty | Clubbing | Clubbing |
| **Initial clinical findings** | Limited motion in the knee and hip joints | Hepatosplenomegaly | - | Cachectic appearance, early teeth loss, multiple nevus, sparse eye brows, limited movement of elbow, kyphoscoliosis | - | Prominent metopic suture | - | Swelling of phalanges  Bowed legs  Broad long bones  Large anterior fontanel | Clubbing, coarse facial features, widely open anterior fontanel and sutures, hypertrichosis, inguinal hernia, large hands and feet  Swelling and pain of phalanges | Clubbing, widely open fontanel, large hands and feet |
| **Additional follow-up clinical findings** | Pain resolved | Bicytopenia and hepatosplenomegaly resolved  Pain during long distance walking | Intermittent limb pain | Severe short stature in adulthood  Anterior curvature of tibia developed Kyphoscoliosis, and joint contractures progressed significantly,  severe bone pain developed at the age of 28 years (regressed after the use of teriparatide) | - | - | - | Swelling of phalanges resolved  Severe short stature in adulthood  Joint contractures developed and progressed with age.  Forward bent posture  Scoliosis developed at the age of 22 years | Swelling of phalanges resolved  Excessive palmar sweating  Enlarged knee joint | Pain in the phalanges  Excessive palmar sweating  Enlarged knee joint |
| **Hematological involvement** | - | Bicytopenia | - | - | - | - | - | - | - | - |
| **Eye problems** | - | - | - | Subcapsular cataract | - | - | - | - | - | - |
| **Hearing loss** | - | - | - | + (12 years) | - |  | - | + |  | - |
| **Renal anomaly** | Mild dilatation in the left renal pelvis | - | Grade 1 echogenicity increase (7.5 years) | - | - | - | - | - | - | - |
| **Cardiac anomaly** | - | -- | - | - | - | - | - | - | - | PDA |
| **Intellectual disability** | - | - | - | - | - | - | - | - | - |  |
| **Fracture history (n) (age)** | + (1)  (18 years) | - | - | + (1) (10 years) | + (1) (12 years) | + (4)  (Between one and 3 months of age) | + (1)  (4 months) | + (2)  (15 and 21 years) | - | - |
| **Other** | Familial Mediterranean fever | - | - | Significantly elevated ALP  (2237u/l)  Hashimoto thyroiditis | Mildly elevated ALP  (240 u/l)  Vitiligo | Significantly elevated ALP  (1297u/l)  Anal atresia  Biotinidase deficiency | Significantly elevated ALP  (910u/l) | Significantly elevated ALP  (2727u/l) | - | - |
| **Radiological findings** |  |  |  |  |  |  |  |  |  |  |
| **Initial radiological findings** | Mild to moderate sclerosis of long bone diaphysis  Diaphyseal widening  Cortical thickening  Coxa valga | Moderate sclerosis of long bone diaphysis  Diaphyseal widening  Cortical thickening | Mild sclerosis of long bone diaphysis  Diaphyseal widening  Cortical thickening | Coarse trabecular pattern of long bones  Moderate sclerosis of long bones  Femoral and tibial bowing  Periosteal hyperostosis  Diaphyseal widening | Coarse trabecular pattern of long bones  Periosteal hyperostosis | Osteopenia  Coarse trabecular pattern of long bone diaphysis  Periosteal hyperostosis  Diaphyseal widening | Osteopenia  Coarse trabecular pattern of long bone diaphysis  Periosteal hyperostosis  Diaphyseal widening | Osteopenia  Coarse trabecular pattern of long bone diaphysis  Periosteal hyperostosis  Femoral bowing  Diaphyseal widening | Cranial ossification defect with wormian bones  Diaphyseal widening  Cortical thickening  Periosteal hyperostosis | Cranial ossification defect with wormian bones  Diaphyseal widening  Cortical thickening  Periosteal hyperostosis |
| **Additional follow-up radiological findings** | - | - | - | Osteosclerosis became in a patchy pattern and coarse trabeculation became more evident  Kyphoscoliosis, tibial bowing progressed significantly | - | - | - | Patchy osteosclerosis developed, coarse trabecular pattern became more evident with age  Scoliosis developed | Cranial ossification defect resolved  Cortical thickening and periosteal hyperostosis  became less evident  Acro-osteolysis developed | Cranial ossification defect resolved  Cortical thickening and periosteal hyperostosis  became less evident  Acro-osteolysis developed |
| **DEXA (initial)** | -0.4 | +1.0 | -0.1 | -4.6 | -2.8 | NA | NA | -2.85 | NA | NA |
| **DEXA (last)** | -0.3 | +0.3 | NA | -3.1 | -2.3 | NA | NA | -2.0 | NA | NA |
| **Treatment and surgery history** | Colchicine | Prednisolone | - | Pamidronate, teriparatide | - | Pamidronate | Pamidronate | Calcitonine, pamidronate | - | - |

**Table S2: Continuous**

| **Family Number** | **14** | **15** | | **16** | | **17** | **18** | **19** | | |
| --- | --- | --- | --- | --- | --- | --- | --- | --- | --- | --- |
| **Patient Number** | **21** | **22** | **23** | **24** | **25** | **26** | **27** | **28** | **29** | **30** |
| **Diagnosis** | **Primary hypertrophic osteoarthropathy-1** | | | | | | | **Primary hypertrophic osteoarthropathy-2** | | |
| **Gender(F/M)** | M | F | F | M | M | F | M | M | M | M |
| **Parental**  **Consanguinity** | - | + | | + | | + | - | + | | |
| **Age at admission(years)** | 39 | 6.8 | 12.7 | 12.7 | 17 | 22 | 17 | 21 | 19 | 7 |
| **Age at last examination** | 39 | 13.8 | 18 | 15 | 20 | 24 | 17 | 24 | 21 | 7 |
| **Height SDS at admission** | -0.68 | 0.86 | -1.72 | -0.43 | 0.77 | -0.02 | -1.09 | 1 | -0.7 | 0.8 |
| **Height SDS at last examination** | NA | 0.24 | -1.02 | -0.46 | 1.59 | -0.02 | NA | NA | NA | NA |
| **Clinical findings** |  |  |  |  |  |  |  |  |  |  |
| **Admission symptom** | Clubbing, joint pain, excessive sweating | Clubbing, enlarged knee joint | Clubbing, enlarged knee joint | Clubbing, enlarged knee, wrist, ankle joints | Clubbing, enlarged knee, wrist, ankle joints | Clubbing | Clubbing, enlarged knee, wrist, ankle joints | Clubbing, enlarged joints, cutis gyrata | Clubbing, enlarged joints, cutis gyrata | Delayed closure of cranial sutures |
| **Initial clinical findings** | Clubbing, enlarged hands and feet, pachydermia, enlarged knee joint | Clubbing, enlarged hands and feet, enlarged knee joint, pachydermia  Excessive palmar /plantar sweating | Clubbing, enlarged hands and feet, enlarged knee joint, pachydermia  Excessive palmar /plantar sweating | Clubbing, enlarged knee, wrist, ankle joints  Excessive palmar /plantar sweating, pachydermia | Clubbing, enlarged knee, wrist, ankle joints  Excessive palmar /plantar sweating, enlarged hands and feet, pachydermia | Clubbing, excessive palmar /plantar sweating, pachydermia | Clubbing, enlarged knee, wrist, ankle joints Asthenic habitus | Coarse face, enlarged joints, clubbing, seborrheic hyperplasia, excessive palmar /plantar sweating, pachydermia | Coarse face, enlarged joints, clubbing, seborrheic hyperplasia, excessive palmar /plantar sweating, pachydermia | Delayed closure of cranial sutures |
| **Additional follow-up clinical findings** | - | - | - | Joint pain developed | Joint pain developed | - | - | - | - | - |
| **Hematological involvement** | - | - | - | - | - | - | - | - | - | - |
| **Eye problems** | - | - | - | - | - | - | - | - | - | - |
| **Hearing loss** | - | - | - | - | - | - | - | - | - | - |
| **Renal anomaly** | - | - | - | - | - | - | - |  |  |  |
| **Cardiac anomaly** | - | Mild mitral regurgitation | Mild mitral regurgitation, MVP | - | - | - | - | - | - | - |
| **Intellectual disability** | - | - | - | - | - | - | - | - | - | - |
| **Fracture history (n) (age)** | - | - | - | - | - | - | - | - | - | - |
| **Other** | - | - | - | - | - | - | - | - | Cranial imaging: mild dilation of lateral ventricles |  |
| **Radiological findings** |  |  |  |  |  |  |  |  |  |  |
| **Initial radiological findings** | Diaphyseal widening  Cortical thickening  Periosteal hyperostosis | Cortical thickening  Periosteal hyperostosis  Acro-osteolysis | Cortical thickening  Periosteal hyperostosis  Acro-osteolysis | Cortical thickening | Cortical thickening | Cortical thickening  Periosteal hyperostosis  Acro-osteolysis | Cortical thickening  Periosteal hyperostosis  Acro-osteolysis | Cortical thickening  Periosteal hyperostosis | Cortical thickening  Periosteal hyperostosis | Periosteal hyperostosis |
| **Additional follow-up radiological findings** | - | - | - | - | - | - | - | - | - | - |
| **DEXA (initial)** | NA | 0.4 | 1.4 | NA | NA | 0.6 | NA | NA | NA | NA |
| **DEXA (last)** | NA | NA | NA | NA | NA | NA | NA | NA | NA | NA |
| **Treatment and surgery history** | - | - | - | - | - | - | - | - | - | - |

**Table S2: Continuous**

| **Family Number** | **20** | **21** | **22** | **23** |
| --- | --- | --- | --- | --- |
| **Patient Number** | **31** | **32** | **33** | **34** |
| **Diagnosis** | **Trichothiodystrophy** | **Melorheostosis** | **Lenz-Majewski hyperostostic dwarfism** | **Prenatal Caffey disease** |
| **Gender(F/M)** | F | M | F | F |
| **Parental**  **Consanguinity** | - | - | - | - |
| **Age at admission(years)** | 3.8 | 0.7 | 0.5 | 27GW |
| **Age at last examination** | 9.1 | 10.5 | 0.5 | NA |
| **Height SDS at admission** | -4.41 | 0.15 | -4.02 | 0.01 |
| **Height SDS at last examination** | -4.52 | -1.55 | NA | NA |
| **Clinical findings** |  |  |  |  |
| **Admission symptom** | Neuromotor retardation, ichthyosis brittle and sparse hair | Contracture of phalanges | Growth retardation, feeding difficulty | Prenatal shortened extremities, scalp edema |
| **Initial clinical findings** | Ichthyosis brittle and sparse hair, hemangioma, limited movement of elbow, hip, and knee | Hemihypertrophy of right lower limb  Camptodactyly of right 3^rd^ to 5^th^ phalanges  Limited movement of right elbow  Right pes cavus | Macrocephaly, flattened and broad nasal bridge, hypertelorism, anteverted nostrils, and micrognathia, hypotonia, sagging and wrinkled skin,  severe brachydactyly, partial syndactyly, rocker bottom feet | Low-set ears, long philtrum, thin lips, microretrognathia, short neck, hydrops fetalis, polyhydramnios, narrow thorax, short extremities  low-set |
| **Additional follow-up clinical findings** | Walking impairment at the age of 8.5 years | Scoliosis and waddling gait developed due to limb asymmetry | - | - |
| **Hematological involvement** | Neutropenia | - | - | - |
| **Eye problems** | - | - | - | NA |
| **Hearing loss** | - | - | - | NA |
| **Renal anomaly** | Right ectopic kidney, right duplicated collecting system | - | - | - |
| **Cardiac anomaly** | - | - | - | - |
| **Intellectual disability** | - | - | Developmental delay | NA |
| **Fracture history (n) (age)** | - | - | - | - |
| **Other** | Cranial imaging: Hypomyelination | - | - | - |
| **Radiological findings** |  |  |  |  |
| **Initial radiological findings** | Moderate sclerosis of cranium, vertebra, pelvis, and clavicles  Coxa valga | Dripping wax-like sclerosis on pelvis, right metacarpals and phalanges, bilateral metatarsals, epiphysis of bilateral proximal tibia, proximal and distal femur, proximal humerus | Moderate sclerosis of long bones diaphysis  Short phalanges, hypoplasia of 5^th^ metacarpal and metatarsal bones | Periosteal hyperostosis  of ribs and long bones  Irregular cortical densities and a double  contour of diaphyseal cortex of long bones |
| **Additional follow-up radiological findings** | Mild acetabular irregularity, narrowed acetabular joint space developed | Dripping wax-like sclerosis showed progression  Scoliosis developed | - | - |
| **DEXA (initial)** | +4.9 | NA | NA | NA |
| **DEXA (last)** | +4.3 | 0.1 | NA | NA |
| **Treatment and surgery history** | - | Achillotomy | - | - |

ALP: Alkaline phosphatase; DEXA: dual-energy X-ray absorptiometry; F: female; MVP: Mitral valve prolapsus; PDA: patent ductus arteriosus; M:male; NA: not available; OSAS: obstructive sleep apnea syndrome; SDS: standard deviation score
